# Effect of hypoalbuminemia on short-term outcomes after colorectal cancer surgery: A propensity score matching analysis

**DOI:** 10.3389/fnut.2022.925086

**Published:** 2022-08-29

**Authors:** Bing Kang, Zhi-Qiang Zhao, Xiao-Yu Liu, Yu-Xi Cheng, Wei Tao, Zheng-Qiang Wei, Dong Peng

**Affiliations:** ^1^Department of Gastrointestinal Surgery, The First Affiliated Hospital of Chongqing Medical University, Chongqing, China; ^2^Department of Clinical Nutrition, The First Affiliated Hospital of Chongqing Medical University, Chongqing, China; ^3^Department of General Surgery, Qijiang Hospital of the First Affiliated Hospital of Chongqing Medical University, Chongqing, China

**Keywords:** colorectal cancer, hypoalbuminemia, outcomes, complications, surgery

## Abstract

**Purpose:**

The purpose of our study was to investigate the effect of pre-operative hypoalbuminemia on the short-term outcomes after primary colorectal cancer (CRC) surgery.

**Materials and methods:**

The retrospective study enrolled CRC patients who underwent primary surgery from January 2011 to December 2021 in a single teaching hospital. The short-term outcomes were compared between the hypoalbuminemia group and the normal group using propensity score matching (PSM). Univariate and multivariate logistic regression analyses were used for analyzing independent predictors of overall complications and major complications.

**Results:**

A total of 7,072 patients from a single center were enrolled in this study. There were 1,078 (15.2%) patients in the pre-operative hypoalbuminemia group and 5,994 (84.8%) patients in the normal pre-operative albumin group. After 1:1 PSM, there were 1,028 patients in the hypoalbuminemia group and 1,028 patients in the normal group. No significant differences were found in baseline information between the two groups after PSM. In terms of short-term outcomes, the hypoalbuminemia group had a longer operation time (*p* = 0.003), greater volume of blood loss (*p* = 0.036), longer hospital stays (*p* < 0.01), higher proportion of overall complications (*p* = 0.003), major complications (*p* = 0.016), higher incidence of pneumonia and abdominal infection (*p* = 0.001) than the normal group after PSM. Furthermore, hypoalbuminemia was an independent predictor for overall complications (*p* = 0.008) and major complications (*p* = 0.016).

**Conclusion:**

Pre-operative hypoalbuminemia increased overall complications and major complications after primary CRC surgery. Furthermore, hypoalbuminemia was an independent predictor for overall complications and major complications.

## Introduction

Colorectal cancer (CRC) is the third most commonly occurring cancer and the second leading cause of cancer-related mortality worldwide (1–3). It is estimated that approximately 147,950 new CRC cases would be diagnosed, and 53,200 individuals would die of this disease in 2020 (4, 5). Although there have been many treatment methods for CRC including endoscopic mucosal resection (EMR), endoscopic submucosal dissection (ESD), surgery, cytotoxic chemotherapy, radiotherapy, and biologic therapy such as antibodies to cellular growth factors, immunotherapy, and combinations of methods (6–8), surgical resection remains the main curative option for both colon and rectal cancer (9–11).

Previous studies have shown that the rates of malnutrition in CRC patients ranged from 20 to 37%, depending on the tool used to assess nutritional status (12, 13). Pre-operative malnutrition is associated with higher post-operative morbidity, mortality, and length of hospital stay (14). Albumin has been used as a nutritional and inflammatory indicator (15) and pre-operative hypoalbuminemia was associated with post-operative complications, mortality, overall survival (OS), and cancer-specific survival (CSS) (16–18).

Hu et al. (19) previously reported an effect of pre-operative mild hypoalbuminemia on post-operative complications in CRC patients using propensity score matching (PSM), however, it depended on the American College of Surgeons-National Surgical Quality Improvement Program (ACS-NSQIP) database which lacks some important data such as tumor stage, operative time, the volume of blood loss, and length of hospital stay which are crucial in analyzing the effect of hypoalbuminemia on prognosis. Therefore, the purpose of our study was to investigate the effect of pre-operative hypoalbuminemia on short-term outcomes after primary CRC surgery using PSM.

## Materials and methods

### Setting

We retrospectively enrolled patients who underwent primary CRC surgery from January 2011 to December 2021 in a single teaching hospital. The study was approved by the ethics committee of the First Affiliated Hospital of Chongqing Medical University (2022-K205), and informed consent was obtained from all participants.

### Study population selection

All enrolled patients underwent radical CRC resection (total mesorectal excision or complete mesocolic excision) by experienced surgeons according to the clinical guidelines, and the pathologic examination confirmed R0 resection. The exclusion criteria were as follows: (1) recurrent CRC surgery; (2) non-R0 CRC surgery, which was confirmed according to the pathologic examination; (3) incomplete baseline information; (4) incomplete information on albumin, hemoglobin, and lymphocyte levels.

### Covariates

Data were collected through the inpatient system, outpatient system, and telephone review. The baseline information included serum albumin level, age, sex, body mass index (BMI), smoking, drinking, hypertension, type 2 diabetes mellitus (T2DM), coronary heart disease (CHD), chronic kidney disease (CKD), chronic liver disease (CLD), surgical history, laparoscopy, serum hemoglobin and lymphocyte levels, tumor size, tumor location, and tumor stage. Blood for testing was collected in the morning after 8 h of fasting in the supine position on the first day after admission.

The short-term outcomes studies were operation time, the volume of blood loss, hospital stay, blood transfusion, and overall complications (including anastomotic leakage, pneumonia, wound infection, lymphatic fistula, intestinal obstruction, venous thrombosis, abdominal infection, 30-day death, and other complications), major complications. The tumor stage was diagnosed according to the AJCC 8th Edition (20). Pre-operative serum albumin < 35 g/L was considered hypoalbuminemia. The severity of post-operative complications was defined according to the Clavien-Dindo classification (21), and ≥III classification, including requiring surgical, endoscopic, or radiological intervention; life-threatening complications (central nervous system complications: brain hemorrhage, ischemic stroke, and subarachnoid bleeding, but excluding transient ischemic attacks); requiring intermediate care (IC)/intensive care unit (ICU) management and death were considered major complications (21, 22).

### Statistical analysis

Propensity score matching was conducted between the hypoalbuminemia group and the normal group to minimize the bias of baseline information. Nearest neighbor matching was performed without replacement at a 1:1 ratio and a caliper width with a.01 standard deviation was specified. The baseline information was matched including age, sex, BMI, smoking, drinking, hypertension, T2DM, CHD, CKD, CLD, surgical history, laparoscopy, serum hemoglobin and lymphocyte levels, tumor size, tumor location, and tumor stage.

For statistical analysis, SPSS 22 analysis software was used. Continuous variables are expressed as the mean ± standard deviation (SD) and an independent-sample *t*-test was used to compare the difference between the hypoalbuminemia group and the normal group. Categorical variables are expressed as absolute values and percentages, and the Chi-square test or Fisher’s exact test was used to compare the differences between the hypoalbuminemia group and the normal group. Univariate and multivariate logistic regression analyses were performed to identify independent predictive factors for overall complications and major complications. A bilateral *p*-value < 0.05 was considered statistically significant.

## Results

**FIGURE 1:**
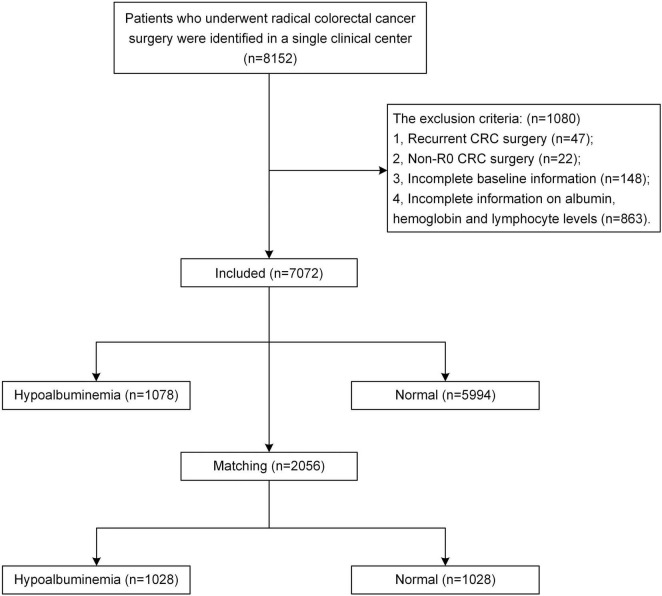
Flow chart of patient selection.

According to the inclusion and exclusion criteria, a total of 7,072 patients with CRC from a single center were enrolled. There were 1,078 (15.2%) patients in the pre-operative hypoalbuminemia group and 5,994 (84.8%) patients in the normal pre-operative albumin level group. After 1:1 PSM, 1028 patients with pre-operative hypoalbuminemia and 1,028 without pre-operative hypoalbuminemia were included in this study. The flow chart of patient selection is shown in Figure 1.

### Baseline information

Baseline information was compared between the hypoalbuminemia group and the normal group. The hypoalbuminemia group had an older age (*p* < 0.01), lower BMI (*p* < 0.01), lower proportion of CHD (*p* < 0.01), lower proportion of laparoscopic CRC surgery (*p* < 0.01), lower serum hemoglobin level (*p* < 0.01), fewer serum lymphocyte (*p* < 0.01), larger tumor size (*p* < 0.01), and higher portion of colon cancer (*p* < 0.01) than the normal group before PSM. However, there were no significant differences in age, CHD, laparoscopy, hemoglobin, lymphocytes, tumor size, tumor location, or tumor stage between the two groups after PSM (*p* > 0.05) (Table 1).

**TABLE 1:** Baseline characteristics before and after PSM.

Characteristics	Before PSM			After PSM		
	Hypoalbuminemia (1078)	Normal (5994)	*P*-value	Hypoalbuminemia (1028)	Normal (1028)	*P*-value
Albumin	31.0 ± 3.1	41.3 ± 4.2	<0.01*	31.1 ± 3.0	39.8 ± 4.3	<0.01*
Age (year)	67.8 ± 12.4	61.9 ± 12.1	<0.01*	67.5 ± 12.4	67.7 ± 11.6	0.753
Sex			0.993			0.964
Male	632 (58.6%)	3515 (58.6%)		601 (58.5%)	602 (58.6%)	
Female	446 (41.4%)	2479 (41.4%)		427 (41.5%)	426 (41.4%)	
BMI (kg/m^2^)	21.6 ± 3.3	22.8 ± 3.1	<0.01*	21.6 ± 3.3	21.7 ± 3.0	0.661
Smoking	410 (38.0%)	2226 (37.1%)	0.575	399 (38.8%)	394 (38.3%)	0.821
Drinking	327 (30.3%)	1832 (30.6%)	0.880	314 (30.5%)	311 (30.3%)	0.886
Hypertension	287 (26.6%)	1500 (25.0%)	0.266	272 (26.5%)	273 (26.6%)	0.960
T2DM	139 (12.9%)	668 (11.1%)	0.096	132 (12.8%)	138 (13.4%)	0.695
CHD	77 (7.1%)	241 (4.0%)	<0.01*	74 (7.2%)	68 (6.6%)	0.602
CKD	199 (18.5%)	1027 (17.1%)	0.290	190 (18.5%)	201 (19.6%)	0.536
CLD	27 (2.5%)	189 (3.2%)	0.255	27 (2.6%)	26 (2.5%)	0.889
Surgical history	273 (25.3%)	1472 (24.6%)	0.591	256 (24.9%)	277 (26.9%)	0.291
Laparoscopy	790 (73.3%)	5381 (89.8%)	<0.01*	777 (75.6%)	769 (74.8%)	0.683
Hemoglobin, g/L	103.3 ± 22.9	124.3 ± 22.7	<0.01*	104.6 ± 22.4	104.4 ± 24.4	0.908
Lymphocyte, 10^9^	1.2 ± 0.5	1.5 ± 0.6	<0.01*	1.2 ± 0.5	1.2 ± 0.5	0.720
Tumor size			<0.01*			0.505
<5 cm	449 (41.7%)	3820 (63.7%)		439 (42.7%)	454 (44.2%)	
≥5 cm	629 (58.3%)	2174 (36.3%)		589 (57.3%)	574 (55.8%)	
Tumor location			<0.01*			0.430
Colon	710 (65.9%)	2625 (43.8%)		666 (64.8%)	683 (66.4%)	
Rectum	368 (34.1%)	3369 (56.2%)		362 (35.2%)	345 (33.6%)	
Tumor stage			<0.01*			0.666
I	119 (11.0%)	1206 (20.1%)		116 (11.3%)	127 (12.4%)	
II	524 (48.6%)	2307 (38.5%)		495 (48.2%)	468 (45.5%)	
III	375 (34.8%)	2165 (36.1%)		360 (35.0%)	375 (36.5%)	
IV	60 (5.6%)	316 (5.3%)		57 (5.5%)	58 (5.6%)	

Variables are expressed as the mean ± SD, n (%), *P-value < 0.05.

T2DM, type 2 diabetes mellitus; BMI, body mass index; PSM, propensity score matching; CHD, coronary heart disease; CKD, chronic kidney disease; CLD, chronic liver disease.

### Short-term outcomes

Table 2 shows the differences in short-term outcomes, including operation time, the volume of blood loss, hospital stay, blood transfusion, overall complications, major complications, 30-day death, and different types of post-operative complications between the hypoalbuminemia group and the normal group before and after PSM. The hypoalbuminemia group had a longer operation time (*p* = 0.015), greater volume of blood loss (*p* < 0.01), longer hospital stays (*p* < 0.01), higher proportion of blood transfusion (*p* < 0.01), overall complications (*p* < 0.01), major complications (*p* < 0.01), and 30-day death (*p* < 0.01) than the normal group before PSM. In terms of post-operative complications, the hypoalbuminemia group had more pneumonia (*p* < 0.01), wound infection (*p* = 0.005), venous thrombosis (*p* = 0.02), abdominal infection (*p* < 0.01), and other complications (*p* = 0.001) than the normal group. After PSM, the hypoalbuminemia group still had a longer operation time (*p* = 0.003); greater volume of blood loss (*p* = 0.036); longer hospital stay (*p* < 0.01); higher proportion of overall complications (*p* = 0.003) and major complications (*p* = 0.016); and a greater incidence of pneumonia (*p* = 0.001) and abdominal infection (*p* = -0.02) than the normal group.

**TABLE 2:** Short-term outcomes before and after PSM.

Characteristics	Before PSM			After PSM		
	Hypoalbuminemia (1078)	Normal (5994)	*P*-value	Hypoalbuminemia (1028)	Normal (1028)	*P*-value
Operation time (min)	232.0 ± 88.1	225.2 ± 83.6	0.015*	231.9 ± 88.3	220.9 ± 76.9	0.003*
Blood loss (mL)	132.4 ± 229.4	99.7 ± 143.3	<0.01*	133.5 ± 233.3	113.9 ± 159.4	0.036*
Hospital stay (day)	12.8 ± 8.6	10.9 ± 8.0	<0.01*	12.7 ± 8.6	11.4 ± 6.9	<0.01*
Blood transfusion	60 (5.6%)	97 (1.6%)	<0.01*	55 (5.4%)	44 (4.3%)	0.257
Overall complications	341 (31.6%)	1168 (19.5%)	<0.01*	323 (31.5%)	263 (25.6%)	0.003*
Major complications	52 (4.8%)	148 (2.5%)	<0.01*	50 (4.9%)	29 (2.8%)	0.016*
30-day death	12 (1.1%)	12 (0.2%)	<0.01*	10 (1.0%)	6 (0.6%)	0.315
Anastomotic leakage	27 (2.5%)	157 (2.6%)	0.828	26 (2.5%)	18 (1.8%)	0.223
Pneumonia	80 (7.4%)	143 (2.4%)	<0.01*	75 (7.3%)	39 (3.8%)	0.001*
Wound infection	52 (4.8%)	189 (3.2%)	0.005*	49 (4.8%)	41 (4.0%)	0.388
Lymphatic fistula	10 (0.9%)	32 (0.5%)	0.121	10 (1.0%)	8 (0.8%)	0.636
Intestinal obstruction	22 (2.0%)	115 (1.9%)	0.789	22 (2.1%)	22 (2.1%)	1.000
Venous thrombosis	17 (1.6%)	50 (0.8%)	0.020*	17 (1.7%)	16 (1.6%)	0.861
Abdominal infection	91 (8.4%)	295 (4.9%)	<0.01*	86 (8.4%)	59 (5.7%)	0.020*
Other complications	100 (9.3%)	393 (6.6%)	0.001*	95 (9.2%)	99 (9.6%)	0.763

Variables are expressed as the mean ± SD, n (%), *P-value < 0.05.

PSM, propensity score matching.

### Univariate and multivariate logistic regression analyses of the overall complications

Age (*p* < 0.01, HR = 1.031, 95% CI = 1.022–1.04), surgical methods (*p* < 0.01, HR = 1.745, 95% CI = 1.41–2.158), T2DM (*p* < 0.01, HR = 1.727, 95% CI = 1.323–2.253), CHD (*p* = 0.016, HR = 1.543, 95% CI = 1.082–2.199), CKD (*p* = 0.001, HR = 1.503, 95% CI = 1.19–1.899), hypoalbuminemia (*p* = 0.003, HR = 1.333, 95% CI = 1.2–1.615), volume of blood loss (*p* < 0.01, HR = 1.002, 95% CI = 1.001–1.002), and operation time (*p* < 0.01, HR = 1.003, 95% CI = 1.002–1.005) were predictors in univariate analysis. In multivariate analysis, age (*p* < 0.01, HR = 1.033, 95% CI = 1.023–1.043), surgical methods (*p* < 0.01, HR = 1.631, 95% CI = 1.302–2.042), T2DM (*p* = 0.008, HR = 1.459, 95% CI = 1.105–1.927), hypoalbuminemia (*p* = 0.008, HR = 1.312, 95% CI = 1.075–1.602), blood loss (*p* = 0.009, HR = 1.001, 95% CI = 1–1.001), and operation time (*p* < 0.01, HR = 1.003, 95% CI = 1.002–1.004) were independent predictors for overall complications (Table 3).

**TABLE 3:** Univariate and multivariate logistic regression analysis of the overall complications.

Risk factors	Univariate analysis		Multivariate analysis	
	OR (95% CI)	*P*-value	OR (95% CI)	*P*-value
Age, year	1.031 (1.022–1.040)	<0.01*	1.033 (1.023–1.043)	<0.01*
Surgical methods (open/laparoscopic)	1.745 (1.410–2.158)	<0.01*	1.631 (1.302–2.042)	<0.01*
Sex (male/female)	0.887 (0.730–1.079)	0.230		
BMI, Kg/m^2^	0.983 (0.953–1.013)	0.267		
Hypertension (yes/no)	1.236 (0.999–1.530)	0.051		
T2DM (yes/no)	1.727 (1.323–2.253)	<0.01*	1.459 (1.105–1.927)	0.008*
Surgical history (yes/no)	1.188 (0.958–1.473)	0.117		
Tumor location (colon/rectum)	1.016 (0.830–1.243)	0.877		
Tumor stage (IV/III/II/I)	1.022 (0.901–1.159)	0.737		
Smoking (yes/no)	1.010 (0.830–1.229)	0.922		
Drinking (yes/no)	0.998 (0.811–1.230)	0.988		
CHD (yes/no)	1.543 (1.082–2.199)	0.016*	1.177 (0.811–1.710)	0.391
CKD (yes/no)	1.503 (1.190–1.899)	0.001*	1.091 (0.846–1.405)	0.503
CLD (yes/no)	1.539 (0.876–2.704)	0.134		
Tumor size (≥5/<5), cm	1.097 (0.904–1.332)	0.348		
Albumin (hypoalbuminemia/normal), g/L	1.333 (1.200–1.615)	0.003*	1.312 (1.075–1.602)	0.008*
Hemoglobin, g/L	0.997 (0.992–1.001)	0.097		
Lymphocyte, 10^9^	0.825 (0.676–1.007)	0.058		
Blood loss, mL	1.002 (1.001–1.002)	<0.01*	1.001 (1.000–1.001)	0.009*
Operation time, min	1.003 (1.002–1.005)	<0.01*	1.003 (1.002–1.004)	<0.01*

*P-value < 0.05.

OR, odds ratio; CI, confidence interval; BMI, body mass index; T2DM, type 2 diabetes mellitus; CHD, coronary heart disease; CKD, chronic kidney disease; CLD, chronic liver disease.

### Univariate and multivariate logistic regression analyses of the major complications

In terms of the major complications, age (*p* = 0.005, HR = 1.031, 95% CI = 1.009–1.053), surgical methods (*p* = 0.003, HR = 2.02, 95% CI = 1.271–3.21), tumor location (*p* = 0.019, HR = 0.582, 95% CI = 0.37–0.914), drinking (*p* = 0.048, HR = 1.589, 95% CI = 1.044–2.516), hypoalbuminemia (*p* = 0.017, HR = 1.761, 95% CI = 1.105–2.806), and serum lymphocyte (*p* = 0.045, HR = 0.599, 95% CI = 0.363–0.988) were predictors in univariate analysis. In multivariate analysis, age (*p* = 0.004, HR = 1.032, 95% CI = 1.01–1.054), surgical methods (*p* = 0.001, HR = 2.284, 95% CI = 1.405–3.713), tumor location (*p* = 0.003, HR = 0.49, 95% CI = 0.306–0.785), drinking (*p* = 0.036, HR = 1.648, 95% CI = 1.033–2.63), and hypoalbuminemia (*p* = 0.016, HR = 1.787, 95% CI = 1.116–2.86) were independent predictors for major complications (Table 4).

**TABLE 4:** Univariate and multivariate logistic regression analysis of the major complications.

Risk factors	Univariate analysis		Multivariate analysis	
	OR (95% CI)	*P*-value	OR (95% CI)	*P*-value
Age, year	1.031 (1.009–1.053)	0.005*	1.032 (1.010–1.054)	0.004*
Surgical methods (open/laparoscopic)	2.020 (1.271–3.210)	0.003*	2.284 (1.405–3.713)	0.001*
Sex (male/female)	0.812 (0.509–1.294)	0.380		
BMI, Kg/m^2^	0.944 (0.876–1.016)	0.124		
Hypertension (yes/no)	1.004 (0.603–1.670)	0.988		
T2DM (yes/no)	0.845 (0.417–1.713)	0.641		
Surgical history (yes/no)	1.339 (0.824–2.174)	0.238		
Tumor location (colon/rectum)	0.582 (0.370–0.914)	0.019*	0.490 (0.306–0.785)	0.003*
Tumor stage (IV/III/II/I)	0.745 (0.551–1.006)	0.055		
Smoking (yes/no)	1.088 (0.530–1.322)	0.718		
Drinking (yes/no)	1.589 (1.044–2.516)	0.048*	1.648 (1.033–2.630)	0.036*
CHD (yes/no)	0.711 (0.256–1.972)	0.512		
CKD (yes/no)	1.366 (0.806–2.317)	0.247		
CLD (yes/no)	2.099 (0.738–5.967)	0.164		
Tumor size (≥5/<5), cm	0.914 (0.582–1.436)	0.696		
Albumin (hypoalbuminemia/normal), g/L	1.761 (1.105–2.806)	0.017*	1.787 (1.116–2.860)	0.016*
Hemoglobin, g/L	1.004 (0.994–1.013)	0.451		
Lymphocyte, 10^9^	0.599 (0.363–0.988)	0.045*	0.666 (0.408–1.086)	0.103
Blood loss, mL	1.001 (1.000–1.001)	0.050		
Operation time, min	1.001 (0.997–1.002)	0.747		

*P-value < 0.05.

OR, odds ratio; CI, confidence interval; BMI, body mass index; T2DM, type 2 diabetes mellitus; CHD, coronary heart disease; CKD, chronic kidney disease; CLD, chronic liver disease.

## Discussion

In this study, we found that the hypoalbuminemia group had a longer operation time and hospital stay, a greater volume of blood loss, a higher proportion of overall complications and major complications, and a greater incidence of pneumonia and abdominal infection than the normal group after 1:1 PSM. Hypoalbuminemia was an independent predictor for overall complications and major complications.

Some studies strengthened the correlation of pre-operative hypoalbuminemia with post-operative outcomes in CRC patients (15–19, 23–31), and we summarize them in Table 5. Some of these studies highlighted the prognosis, such as OS (18, 23–25), CSS (18, 23), and relapse-free survival (RFS) (24), and found that hypoalbuminemia was a prognostic factor for the poorer long-term survival of colon and rectal cancer after curative surgery.

**TABLE 5:** Previous studies reporting the Hypoalbuminemia on the outcomes of CRC patients.

Author	Year	Country	Sample size	Cut-off serum albumin (g/L)	Hypoalbuminemia group	Normal group	Patients	Outcomes
Lohsiriwat (16)	2007	Thailand	112	35	48	36	Stage I–IV right-sided CC	Operative time, blood loss, complications, LOS
Lohsiriwat (17)	2008	Thailand	244	35	56	188	Stage I–IV RC	Operative time, blood transfusion, complications, LOS
Sun (23)	2009	China	1,367	35	392	975	Stage I–IV CRC	OS, CSS
Lai (24)	2011	Taiwan	3,732	35	693	3,039	Stage I–III CC	OS, RFS, complications, mortality
Chandrasinghe (25)	2013	Sri Lanka	226	35	45	181	Stage I–IV RC	OS
Ionescu (26)	2013	Romania	252	35	75	177	Stage 0–IV CRC	Complications, LOS, mortality
Montomoli (27)	2015	Denmark	9,339	35	2,927	6,412	Stage I–IV CRC	Mortality
Chiang (15)	2017	Taiwan	3,732	35	731	3,091	Stage I–IV CRC	Complications, mortality
Hardt (28)	2017	Germany	370	35	67	303	Stage 0–IV RC	Complications
Haskins (29)	2017	United States	5,143	35	746	4,397	Stage I–III CC	Complications, mortality
Hu (19)	2019	United States	12,915	35	4,305	8,610	Stage I–IV CRC	Complications, mortality (using PSM)
Almasaudi (18)	2020	United Kingdom	795	35	250	545	Stage I–IV CRC	OS, CSS, complications
Sofić (30)	2021	Germany	107	35	75	32	Stage I–IV CRC	Complications
Yang (31)	2021	Taiwan	106	35	23	83	Stage I–III CRC	LOS

CRC, colorectal cancer; RC, rectal cancer; CC, colon cancer; OS, overall survival; CSS, cancer-specific survival; RFS, relapse-free survival; DFS, disease-free survival; LOS, length of hospital stay; PSM, propensity score matching.

Regarding short-term outcomes, some studies reported that pre-operative hypoalbuminemia was associated with post-operative outcomes following CRC surgery including prolonged length of hospital stay (16, 17, 26, 31), more complications (15, 17–19, 24, 26, 28, 29), and higher incidence of mortality (15, 19, 27, 29). However, most of these studies had small sample sizes, and all of them lacked the matching of confounding factors in basic data which may interfere with the results. Hu et al. (19) previously reported that pre-operative mild hypoalbuminemia affected post-operative complications for CRC patients using PSM, however, it depended on the ACS-NSQIP database which lacked some important data such as tumor stage, operative time, the volume of blood loss, and length of hospital stay which were crucial in analyzing the effect of hypoalbuminemia on prognosis. Interestingly, the study of Sofić et al. (30) showed that pre-operatively measured levels of serum albumin cannot serve as predictors for post-operative complications. Therefore, whether pre-operative hypoalbuminemia can affect short-term outcomes or be an independent predictor for overall complications and major complications after primary radical CRC surgery should be analyzed in a large sample size with less confounding baseline information.

In this study, we analyzed the effect of pre-operative hypoalbuminemia on the short-term outcomes of CRC surgery, furthermore, PSM was conducted to minimize the bias of baseline information. To avoid affecting pre-operative serum albumin levels, commodities, including CKD and CLD were analyzed by PSM between the two groups. We found that pre-operative hypoalbuminemia prolonged the operation time and hospital stays and increased blood loss and the incidence of major complications and pneumonia. Albumin is the most abundant protein in human blood and increasing capillary permeability during inflammation promotes albumin transfer to the interstitial space (32, 33). Hypoalbuminemia was associated with inflammation (32, 34, 35), indicating that in patients with pre-operative inflammation (36), the inflammation might induce more blood loss, increase the difficulty of the operation and prolong the operation time. Furthermore, hypoalbuminemia affects tissue damage and wound healing post-operatively, impairing gastrointestinal function and mobility, and slowing recovery (35, 36), which might lead to a longer hospital stay.

After 1:1 PSM, we found that the hypoalbuminemia group had more overall complications than the normal group. In one study that enrolled 75 patients in the hypoalbuminemia group and 32 patients in the normal group pre-operatively measured levels of serum albumin could not serve as predictors for post-operative complications (30). The sample size of this German study was small, and some bias might exist. In the present study, hypoalbuminemia was found to be an independent predictive factor of overall complications, which was consistent with a previous study (17). A decrease in pre-operative albumin might be associated with inflammation which increases tissue catabolism (37), and patients with colorectal cancer might have accelerated loss of albumin from the gastrointestinal tract (32, 37, 38). Therefore, hypoalbuminemia might increase the incidence of post-operative overall complications.

Regarding major complications, the hypoalbuminemia group had more major complications than the normal group after PSM in this study. Many factors might affect major complications after primary CRC surgery, such as age, pre-operative morbidities, surgical methods, and tumor stage (39). A previous study showed that hypoalbuminemia was an independent risk factor for post-operative high-grade morbidity (28), and Montomoli et al. (27) found that pre-operative hypoalbuminemia increased 30-day mortality following CRC surgery. Our study was consistent with these studies, and we also found that hypoalbuminemia was an independent predictive factor of major complications. Large samples and randomized controlled trials are needed to clarify the mechanisms of this correlation.

The current study analyzed the effect of hypoalbuminemia on short-term outcomes after CRC surgery in relatively big data using PSM. To our knowledge, this was the first study to analyze the effect of pre-operative hypoalbuminemia on short-term outcomes after primary radical CRC surgery in southwestern China.

There are some limitations of our study. First, this was a retrospective single-center study that inevitably had some selection bias. Second, the important inflammation parameter C-reactive protein (CRP) was not a routine test in our patients; we did not include CRP in this study and did not judge the degree of inflammation. Third, we defined hypoalbuminemia according to the lower limit of reference values and did not consider it a continuous variable or classify it into multiple categories, which might affect the results. Multicenter and multigroup (according to different serum albumin levels) trials with long-term prognosis could be conducted further.

In conclusion, pre-operative hypoalbuminemia increases overall complications and major complications after primary CRC surgery. Furthermore, hypoalbuminemia is an independent predictor for overall complications and major complications.

## Data availability statement

The original contributions presented in this study are included in the article/supplementary material, further inquiries can be directed to the corresponding author.

## Ethics statement

The studies involving human participants were reviewed and approved by The First Affiliated Hospital of Chongqing Medical University, 2022-K205. The patients/participants provided their written informed consent to participate in this study.

## Author contributions

BK, X-YL, and DP contributed to the conception and design of the study. Y-XC and WT organized the database. DP finished the statistical analysis. BK and X-YL wrote the first draft of the manuscript. All authors contributed to revising the manuscript, gave final approval of the version to be published, and agreed to be accountable for all aspects of the work.
